# The majority of dorsal spinal cord gastrin releasing peptide is synthesized locally whereas neuromedin B is highly expressed in pain- and itch-sensing somatosensory neurons

**DOI:** 10.1186/1744-8069-8-52

**Published:** 2012-07-09

**Authors:** Michael S Fleming, Daniel Ramos, Seung Baek Han, Jianyuan Zhao, Young-Jin Son, Wenqin Luo

**Affiliations:** 1 Department of Neuroscience, Perelman School of Medicine, University of Pennsylvania, 145 Johnson Pavilion, 3610 Hamilton Walk, Philadelphia, PA, 19104, USA; 2 Shriners Hospitals Pediatric Research Center, Temple University School of Medicine, 3500 N. Broad Street, Philadelphia, PA, 19140-5104, USA; 3 Department of Anesthesiology and Critical Care medicine, Johns Hopkins University School of Medicine, 1721 E. Madison St, Baltimore, MD, 21205, USA

**Keywords:** Gastrin releasing peptide, Neuromedin B, Itch, Dorsal root ganglion, Spinal cord

## Abstract

**Background:**

Itch is one of the major somatosensory modalities. Some recent findings have proposed that gastrin releasing peptide (Grp) is expressed in a subset of dorsal root ganglion (DRG) neurons and functions as a selective neurotransmitter for transferring itch information to spinal cord interneurons. However, expression data from public databases and earlier literatures indicate that *Grp* mRNA is only detected in dorsal spinal cord (dSC) whereas its family member neuromedin B (*Nmb*) is highly expressed in DRG neurons. These contradictory results argue that a thorough characterization of the expression of *Grp* and *Nmb* is warranted.

**Findings:**

*Grp* mRNA is highly expressed in dSC but is barely detectable in DRGs of juvenile and adult mice. Anti-bombesin serum specifically recognizes Grp but not Nmb. Grp is present in a small number of small-diameter DRG neurons and in abundance in layers I and II of the spinal cord. The reduction of dSC Grp after dorsal root rhizotomy is significantly different from those of DRG derived markers but similar to that of a spinal cord neuronal marker. Double fluorescent *in situ* of *Nmb* and other molecular markers indicate that *Nmb* is highly and selectively expressed in nociceptive and itch-sensitive DRG neurons.

**Conclusion:**

The majority of dSC Grp is synthesized locally in dorsal spinal cord neurons. On the other hand, *Nmb* is highly expressed in pain- and itch-sensing DRG neurons. Our findings provide direct anatomic evidence that Grp could function locally in the dorsal spinal cord in addition to its roles in DRG neurons and that Nmb has potential roles in nociceptive and itch-sensitive neurons. These results will improve our understanding about roles of Grp and Nmb in mediating itch sensation.

## Background

Itch is one of the major somatosensory modalities. Pruritogenic stimuli are detected by somatosensory neurons in the dorsal root ganglia (DRG) and trigeminal ganglia, which transmit itch information to the central nervous system (CNS) by synapsing with dorsal spinal cord (dSC) or medulla interneurons [1]. At present, neuronal mechanisms mediating itch sensation are under intensive investigation in order to identify novel targets for itch therapeutics. Some recent studies have proposed that Grp selectively mediates the transmission of itch information from the DRG to the dSC [2,3].

Grp belongs to the mammalian bombesin-like peptide family, which contains two known members: Grp and Nmb. Grp and Nmb share 80% and 70% identity with bombesin, respectively [4], and selectively bind with high affinity to their respective G-protein coupled receptors, gastrin releasing peptide receptor (Grpr) and neuromedin B receptor (Nmbr) [5] . Grp, Nmb and their receptors are broadly expressed in mammals and their functions have been implied in metabolic regulation, stress response, and cancer pathogenesis [6-10]. Interestingly, both Grp and Nmb have also been found to induce itching behaviors [2,3,11].

The hypothesis that Grp functions as a selective neurotransmitter for itch sensation is supported by the complementary expression pattern of *Grp* and *Grpr* in adult mice, as Grp is detected in a subset of small diameter DRG neurons and their nerve terminals innervating the dSC, where *Grpr* is expressed. In addition, co-injection of a Grpr antagonist with Grp negated the pruritogenic effect of Grp. Furthermore, *Grpr* null mice or mice with ablated bombesin-binding dSC neurons exhibited a specific reduced response to pruritogenic compounds [2,3].

Despite strong evidence in support of the function of Grpr in mediating itch sensation, some concerns exist about the expression pattern of Grp in DRG and dSC neurons. First, *in situ* hybridization data from the Allen Spinal Cord Atlas shows that *Grp* is highly expressed in postnatal day 4 (P4) mouse dSC but it is barely detected in DRG neurons [12]. In addition, the antibody used by previous studies to detect Grp was a rabbit polyclonal antibody generated against the bombesin peptide [2,3,13-15]. Given the very high similarity between mouse Grp and Nmb peptide sequences, it is conceivable that this antibody may recognize both peptides. Indeed, *Nmb* is highly expressed in DRG neurons [12]. Lastly, recent physiological, pharmacological, and genetic studies have demonstrated that glutamate functions as a neurotransmitter of itch-sensing neurons to activate dSC neurons, including Grp-responsive dSC neurons [13-16]. Thus, it is necessary to thoroughly re-examine the expression of *Grp* and *Nmb* in DRG and dSC to resolve some current controversies.

In this study, we used a combination of approaches, including *in situ* hybridization, reverse transcriptase PCR (RT-PCR), real time PCR, immunohistochemistry, and dorsal root rhizotomy to investigate the expression of *Grp* and *Nmb* in DRG and dSC neurons. We found that *Nmb* is highly expressed in DRG neurons and the majority of dSC Grp is synthesized locally in spinal cord neurons. In addition, we found that *Nmb* is specifically expressed in a subpopulation of nociceptive and itch sensitive DRG neurons. Our results suggest that Grp has additional local function in the dSC and Nmb may have potential functions in itch- and pain-sensing DRG neurons.

## Results

### Expression of *GRP* mRNA in juvenile and adult mouse

As mentioned above, *Grp* mRNA is found to be highly expressed in P4 mouse dSC but it is barely detectable in DRG neurons using *in situ* hybridization. On the other hand, Grp has been shown to be specifically expressed in a small population of adult itch-sensing mouse DRG neurons using immunohistochemistry, and dSC Grp is suggested to be transported from DRG neurons [2]. At least two possibilities can explain these inconsistent results: one is that they reflect the normal dynamic expression pattern of *Grp* from early postnatal days to adulthood, and the other is that *Grp* is expressed at a low level in DRG neurons and these results reflect the sensitivity or detection threshold of different techniques.

**Figure 1:**
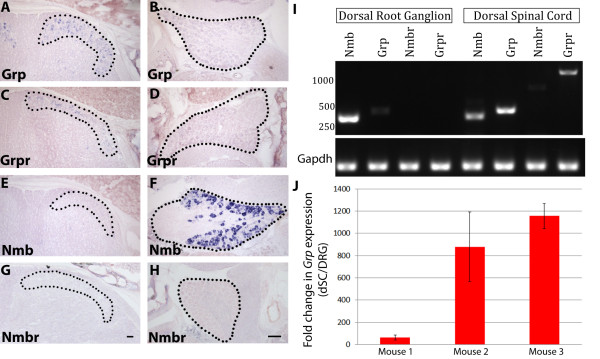
**Expression of*Grp*,*Grpr*,*Nmb*, and*Nmbr*mRNA in dSC and DRG neurons.** (**A**-**H**) *in situ* hybridization with P21 wild type mouse dSC and DRG for *Grp* (**A**-**B**), *Grpr* (**C**-**D**), *Nmb* (**E**-**F**), and *Nmbr* (**G**-**H**). dSC and DRGs are outlined with black dots. Scale bars = 50 μm. (**I**) RT-PCR performed on RNA acutely isolated from wild type adult mouse dSC and DRG. cDNA was amplified with primers specific for *Nmb*, *Grp*, *Nmbr*, and *Grpr*. Lower bands are cDNA amplified with primers specific for *Gapdh*, which served as a loading control. (**J**) Graphical representation of real time RT-PCR for *Grp* performed on dSC and DRG RNA isolated from adult mice (n = 3 animals, 4 replicates per animal). Quantification represents fold difference between dSC Grp relative to that of DRG. Relative abundance of cDNA was calculated in comparison to Gapdh housekeeping gene by the 2^(−ΔΔCт) method (see details in method).

To address these two different possibilities, we first characterized the expression pattern of *Grp* mRNA in juvenile and adult mice using *in situ* hybridization, RT-PCR, and real time PCR. In addition, we investigated the expression pattern of Grpr, the receptor for Grp, and the other mammalian bombesin-related peptide, Nmb, and its receptor, Nmbr. We performed *in situ* hybridization for *Grp*, *Grpr*, *Nmb*, and *Nmbr* on juvenile (P14-P21) wild-type DRG and dSC tissue (n = 3 mice). In agreement with the Allen Spinal Cord Atlas P4 expression data, we found that *Grp* and *Grpr* mRNA could be detected in superficial layers of juvenile mouse dSC, but not in DRG neurons (Figure 1A-1D). In contrast, *Nmb* is highly expressed in ~50% of DRG neurons, but not in dSC (Figure 1E-1F). We could not detect the expression of *Nmbr* in either DRG or dSC at this age, even with very high concentration of antisense probe (Figure 1G-1H). This result is different from the Allen Spinal Cord Atlas P4 data, which shows some expression of *Nmbr* in superficial layers of the dSC. This difference could be due to the dynamic expression of *Nmbr* as mice mature.

To examine the expression pattern of these transcripts by a more sensitive approach, we performed RT-PCR on RNA acutely isolated from either DRGs or dSC of adult wild-type mice (n = 4 mice). In agreement with our *in situ* hybridization data from juvenile mice, we found that *Grp* and *Grpr* could be readily amplified from adult dSC cDNA whereas *Nmb* was robustly amplified from adult DRG cDNA (Figure 1I). Notably, a faint *Grp* band was detected in DRG samples in four out of seven trials. This band was consistently much fainter than the *Grp* product amplified from dSC. As an amplification control, a ubiquitously expressed gene, *Gapdh*, was detected at similar levels in DRG and dSC. The fact that *Grp* cDNA cannot be consistently amplified from freshly isolated adult DRG transcripts indicates that *Grp* is present in DRG neurons at a very low level. This notion is also supported by evidence that *Grp* could not be amplified from a commercially prepared adult DRG cDNA library using PCR, although *Nmb* was readily amplified from the same library (data not shown). To further quantify the relative abundance of *Grp* mRNA in dSC and DRG, we performed real time PCR with dSC and DRG cDNA. We found that *Grp* transcript was present at a much higher concentration in dSC relative to DRG (range = 63-1155 fold difference, n = 3 mice, 4 trials per sample) (Figure 1J). In addition, we noted that faint *Nmb* and *Nmbr* bands were amplified from dSC cDNA (Figure 1I). These slightly different results obtained using *in situ* hybridization and RT-PCR can be explained by the fact that *in situ* hybridization is usually less sensitive compared to reverse transcriptase-and real time PCR. Taken together, our results reveal that *Grp* and *Grpr* mRNA are highly expressed in dSC in both juvenile and adult mice whereas *Nmb* mRNA is highly expressed in DRG neurons. Additionally, *Grp* mRNA is expressed in juvenile and adult mouse DRG neurons at a very low level, which is near the detection threshold of RT-PCR but below that of *in-situ* hybridization.

### Characterization of the specificity of anti-bombesin antiserum

**Figure 2:**
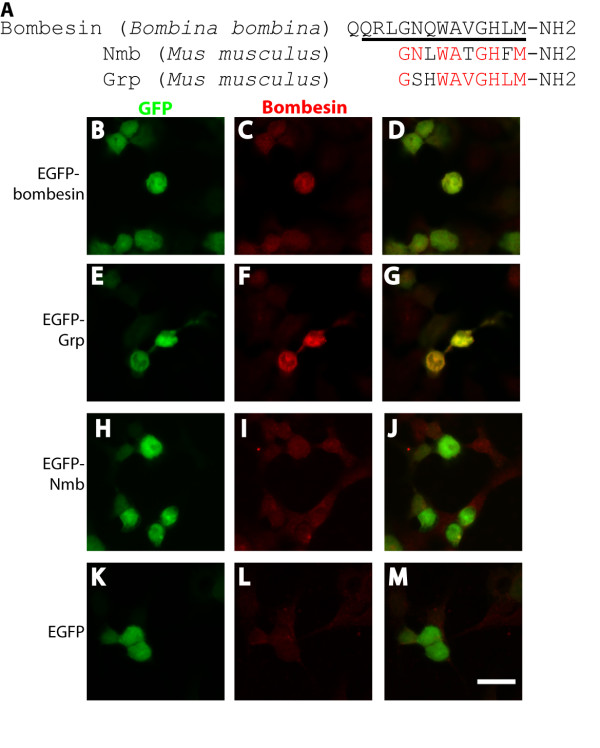
***In vitro*characterization of anti-bombesin antiserum specificity.** (**A**) Alignment of *Bombina bombina* bombesin, *Mus musculus* Grp, and *Mus musculus* Nmb peptide sequences. Black bar underneath the bombesin peptide sequence indicates the immunizing peptide used for developing the rabbit anti-bombesin antiserum. Grp and Nmb residues that share identity with the immunizing peptide are highlighted in red. (**B**-**M**) HEK293 cells were transfected with EGFP-bombesin (**B-D**), EGFP-Grp (**E-G**), EGFP-Nmb (**H-J**), or EGFP alone (**K-M**) and immunostained with anti-bombesin antiserum (red) and anti-GFP antibody (green). Scale bar = 25 μm.

The bombesin family of peptides contains two known family members in mouse: Grp and Nmb. These peptides share 80% and 70% identity, respectively, with the originally characterized member of this family, *Bombina bombina* bombesin (Figure 2A). Previous studies [2,3,13-15] have used a rabbit polyclonal anti-bombesin serum (Immunostar, 20073) to detect the presence of Grp. However, it has not been formally determined whether this anti-bombesin serum selectively recognizes Grp. It is conceivable that this polyclonal antibody detects both Grp and Nmb, given the high similarity among bombesin, Grp, and Nmb. To address this issue, we first characterized the specificity of this polyclonal antibody *in vitro*. In HEK293T cells, we expressed EGFP or EGFP with C terminal fusion of bombesin, Grp, or Nmb. We found that cells expressing EGFP-bombesin and EGFP-Grp were stained by the anti-bombesin antiserum, whereas cells expressing EGFP-Nmb or EGFP alone were not recognized by the antiserum (Figure 2B2M). These findings indicate that the polyclonal antibody used in this and previous studies selectively recognizes Grp but not Nmb, which is quite surprising given the high level of identity between these peptides and the immunizing antigen.

### Characterizing the expression of GRP in DRG neurons and dSC using immunostaining

**Figure 3:**
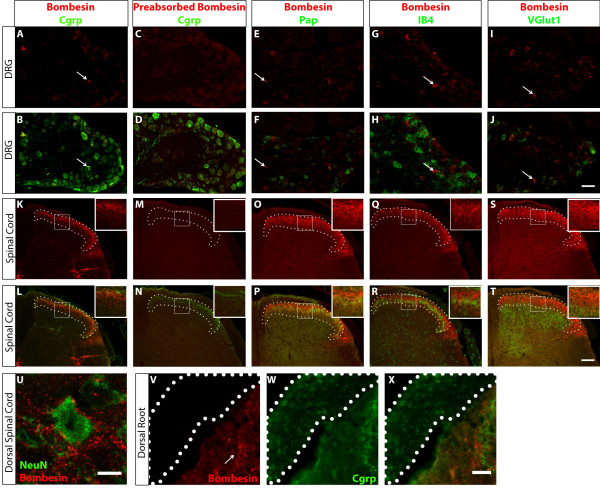
**Characterizing the localization of Grp in DRG, spinal cord, and dorsal root using anti-bombesin antiserum.** (**A**-**B**) DRGs immunostained with antibodies against bombesin (red) and Cgrp (green). Arrow indicates a bombesin^+^;Cgrp^+^ neuron. (**C**-**D**) DRG neurons in adjacent sections are immunostained with anti-bombesin antiserum preabsorbed with purified Grp (red) and anti-Cgrp antibody (green). Preabsorption results in a complete loss of anti-bombesin immunoreactivity. (**E**-**F**) DRG neurons immunostained with antibodies against bombesin (red) and Pap (green). (**G**-**H**) DRG neurons immunostained with anti-bombesin anti-serum (red) and fluorescently-conjugated IB4 (green). (**I**-**J**) DRG neurons immunostained with antibodies against bombesin (red) and VGlut1 (green). Arrows in (**E-J**) indicate bombesin^+^ neurons that are not immunopositive for the costaining marker. (**K**-**L**) dSC section immunostained with antibodies against bombesin (red) and Cgrp (green). The dSC layers positive for bombesin are outlined with white dots and higher magnification of the staining is shown in the inset. (**M**-**N**) dSC section immunostained with anti-bombesin antiserum preabsorbed with purified Grp (red) and anti-Cgrp antibody (green), showing a complete loss of anti-bombesin immunoreactivity. (**O**-**P**) dSC section immunostained with antibodies against bombesin (red) and Pap (green). (**Q**-**R**) dSC section immunostained with anti-bombesin serum (red) and fluorescently-conjugated IB4 (green). (**S**-**T**) dSC section immunostained with antibodies against bombesin (red) and VGlut1 (green). (**U**) A high magnification confocal image of adult dSC immunostained with anti-bombesin (red) and anti-NeuN (green). (**V**-**X**) A higher magnification of wild type adult dorsal root immunostained with antibodies against bombesin (red) and Cgrp (green) reveals the presence of Cgrp, but not Grp, in axons projecting from the DRG to dSC. Dorsal root is outlined by dotted line. Arrow indicates the dSC anti-bombesin immunoreactivity. Scale bars = 50 μm (**A-J**), 100 μm (**K-T**), 5 μm (**U**), 30 μm (**V-X**).

After establishing that the anti-bombesin antiserum selectively recognizes Grp *in vitro*, we next examined the staining pattern of the antiserum *in vivo*. In wild-type P14-P21 mice, we observed that a small number of small-diameter DRG neurons are immunopositive for the anti-bombesin serum (1.79 ± 0.04% of lumbar DRG neurons, n = 3 mice) (Figure 3A-3J). Consistent with previously published results, these Grp^+^ neurons are a subpopulation of peptidergic nociceptors, as they are also Cgrp^+^ (marker of peptidergic nociceptive DRG neurons), but Pap^-^, IB4^-^ (markers of nonpeptidergic nociceptors), and VGlut1^-^ (marker for mechanoreceptors and proprioceptors). This small number of immunopositive neurons is consistent with our finding that *Grp* mRNA is expressed at a very low level in juvenile mouse DRG neurons.

In contrast to DRG neurons, we readily detected the abundant presence of Grp in superficial layers of the dorsal spinal cord. Notably, the immunostaining signal of Grp in dSC is much stronger than that of DRG neurons. We found that Grp is mainly present in layer I and the outer layer of layer II (IIo) of the spinal cord, as the intense Grp signal overlaps with layers innervated by Cgrp, a marker of peptidergic nociceptive DRG afferents terminating in laminae I-IIo of the dorsal horn (Figure 3K3L). Some less intense Grp signal also overlaps with markers of nonpeptidergic nociceptors, such as Pap and IB4, which terminate in lamina IIi of the dorsal horn, and Pkcγ, which is expressed in spinal cord neurons located in lamina IIi and IIIo (Figures 3O3R and 4A4C) [17-19]. However, Grp immunostaining signal does not overlap with VGlut1^+^ puncta, which are formed by Aβ mechanoreceptors innervating layers III-V (Figure 3S3T) [20]. High magnification images of dSC Grp staining show that discrete Grp^+^ puncta are present around NeuN^+^ dSC nuclei, indicating that *Grp* is expressed in dSC neurons (Figure 3U). Taken together, our results reveal that Grp is present at a high level in spinal cord superficial layer neurons.

**Figure 4:**
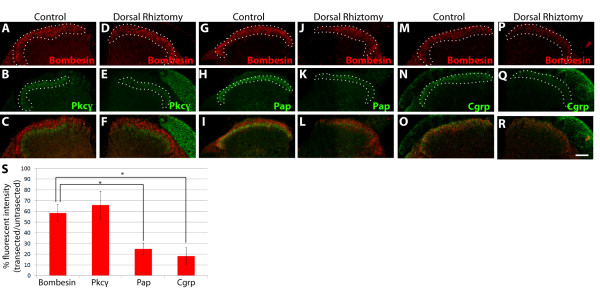
**Dorsal root rhizotomy causes a dramatic loss of Pap and Cgrp but significantly smaller changes of bombesin and Pkcγ in dSC.** L4, L5, and L6 central roots of two month old wild type mice were transected, and mice were examined two weeks following surgery. Sections of the lumbar enlargement were immunostained to examine the loss of proteins and peptides caused by dorsal root axonal degeneration and the contralateral side of the same spinal cord section was used as the control. (**A**-**F**) Sections were immunostained with antibodies against bombesin (red) and Pkcγ (green), a protein highly synthesized in dSC neurons (control side (A-C) and transected side (D-F)). Comparable and modest loss of both bombesin and Pkcγ immunoreactivity are found after the dorsal root rhizotomy. Enhanced green fluorescence and abnormal morphology of the transected dorsal root were also noted, which may be caused by axonal inflammation after injury. (**G**-**L**) Sections immunostained with antibodies against bombesin (red) and Pap (green). As noted, dSC Pap is dramatically decreased after the dorsal root rhizotomy. (**M**-**R**) Sections immunostained with antibodies against bombesin (red) and Cgrp (green), another marker derived from DRG neurons. Similar to Pap, dSC Cgrp is also greatly reduced following the dorsal root rhizotomy. Scale bar = 100 μm. (**S**) Quantification and statistical analysis of dynamic changes of dSC Grp, Pkcγ, Pap, and Cgrp following the dorsal root rhizotomy. The loss of dSC Grp after dorsal root rhizotomy is significantly different from those of Pap and Cgrp (P < 0.01), and the same is true for dSC Pkcγ. In contrast, no significant differences are found between Grp and Pkcγ (P = 0.44) or Pap and Cgrp (P = 0.25).

To further confirm the specificity of immunoreactivity in dSC and DRG, we preabsorbed anti-bombesin antiserum with either bombesin or Grp peptide, and used the supernatant to stain wild type mouse DRGs and spinal cord (n = 3). We found a loss of Grp immunostaining in both dSC and DRG after preabsorbing the antiserum, suggesting that the immunoreactivity we detected in these regions was not due to nonspecific antibodies in the antiserum (Figure 3C-3D and 3M-3N). Unfortunately, the *Grp* null mouse, which would definitively address the *in vivo* specificity of this anti-bombesin serum, is not currently available to us.

### Source of dSC GRP

Even though Grp is present at a high level in layers I and II of the spinal cord, its source is still unclear. It could be synthesized locally in dorsal spinal cord neurons, as suggested by the *Grp* mRNA expression pattern observed in the Allen Spinal Cord Atlas and our own results, or it could be synthesized in and transported from DRG neurons, as previously proposed [2]. Interestingly, Grp is barely detected in dorsal root axons with this anti-bombesin serum (Figure 3V3X). In contrast, we found that Cgrp, Pap, and IB4, which are known markers carried by nociceptive afferents, intensely stain dorsal root axons (Figure 3U3W and data not shown). These findings suggest that the amount of GRP transported from DRG neurons to dSC, if any, is below the detection threshold of our method. So far, our data support that most dSC Grp is synthesized in dSC neurons because: 1) abundant *Grp* transcript is detected in dSC using *in-situ* hybridization and RT-PCR; 2) consistent with the mRNA distribution, a high level of Grp is detected in dSC by immunostaining; 3) a very low level of *Grp* transcript is detected in DRG neurons; 4) a small number of Grp^+^ neurons are present in DRGs; and 5) Grp is barely detected in dorsal root axons.

To further determine the source of dSC Grp, we performed immunostaining on spinal cord following a unilateral dorsal rhizotomy of L4/L5/L6 dorsal roots of adult mice (n = 3). If the primary source of dSC Grp is due to its synthesis in and release from somatosensory afferents, we predict that immunostaining signal for dSC Grp would be greatly decreased following the dorsal rhizotomy. However, if the primary source of dSC Grp is spinal cord neurons, the change in immunostaining signal for dSC Grp following dorsal root rhizotomy should not be as dramatic. We sacrificed the animals two weeks after rhizotomy to ensure that robust degeneration of dorsal root axons had occurred. Spinal cord sections were immunostained with anti-bombesin serum and other antibodies marking DRG sensory axons or spinal cord neurons. The change in immunofluorescence intensity on the transected side was normalized to that of the control side (See methods for details). We found that following L4/L5/L6 dorsal root transection, the intensity of bombesin immunoreactivity is decreased (58.52 ± 8.00% fluorescent intensity, transected/control). Importantly, this decrease is not significantly different from the change of immunoreactivity of Pkcγ (65.91 ± 12.78% fluorescent intensity transected/control), a protein known to be highly expressed in dorsal spinal cord neurons (P = 0.44). The modest reduction of dSC Pkcγ and Grp could be due to the loss of DRG neuron derived Pkcγ and Grp and the denervation response of dSC neurons [21,22]. On the other hand, the immunofluorescence intensity of dSC Pap (24.95 ± 5.58% fluorescent intensity transected/control) and Cgrp (18.25 ± 7.84% fluorescent intensity transected/control), markers primarily transported from nonpeptidergic and peptidergic nociceptors, were dramatically reduced (Figure 4A4S). Remarkably, the signal reduction of Grp and Pkcγ are significantly different from that of Cgrp and Pap (P < 0.01). Taking all findings together, we conclude that the majority of dSC Grp is synthesized locally.

### *Nmb* is mainly expressed in small-diameter DRG neurons

Though *Grp* is only expressed in a few DRG neurons, the other mammalian bombesin-like peptide, *Nmb*, is highly expressed (Figure 1F). This specific expression pattern suggests that Nmb may play a role in somatosensation. Indeed, administration of Nmb has been shown to induce scratching behavior, which can be blocked by administration of an Nmbr antagonist [11]. To further determine a role for Nmb in the itch pathway or other somatosensory modalities, it is important to characterize which subpopulation of DRG neurons expresses *Nmb*. Since there is no commercially available antibody that can reliably and specifically detect Nmb (assay with EGFP-Nmb fusion protein in HEK293 cells, data not shown), we performed double fluorescent *in situ* hybridization (FISH) and FISH combined with immunohistochemistry to establish the molecular profile of Nmb^+^ DRG neurons in P21 mice.

**Figure 5:**
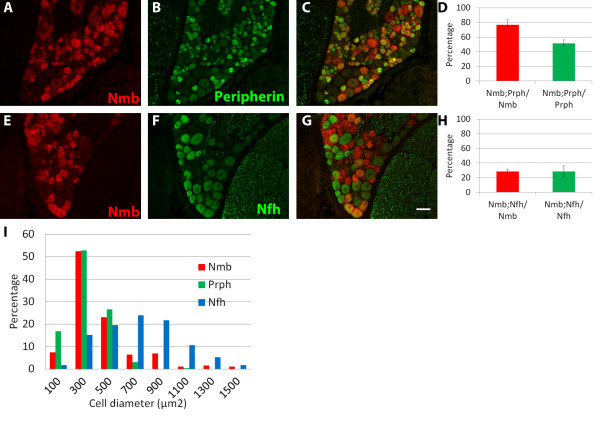
***Nmb*is expressed in small and large diameter DRG neurons.** (**A**-**D**) FISH for *Nmb* (red) and immunostaining for peripherin (green), an intermediate filament that is present in small diameter DRG neurons. Histogram shows the percentage of Nmb^+^ neurons that express peripherin (red bar) and the percentage of Peripherin^+^ neurons that express Nmb (green bar). Mean ± SEM. (**E**-**H**) FISH for *Nmb* (red) and immunostaining for NF200 (green), a marker of large diameter myelinated neurons, and quantification of overlapping expression. Scale bar = 50 μm (**I**) Distribution of soma sizes of Nmb^+^, Peripherin^+^, and NF200^+^ DRG neurons, in 100 μm^2^ bins.

We first examined whether *Nmb* is expressed in large- or small-diameter DRG neurons. DRG neurons with different soma sizes have different developmental origins, molecular profiles, physiological properties, and functional modalities. Small-diameter, unmyelinated or thinly-myelinated DRG neurons, which express the intermediate filament peripherin, are mostly nociceptors. On the other hand, medium- to large-diameter, highly-myelinated DRG neurons, which express the intermediate filament neurofilament heavy chain (Nfh), are mostly mechanoreceptors and proprioceptors. To characterize the size distribution of Nmb^+^ neurons, we performed FISH for *Nmb* and combined it with immunostaining for either peripherin or Nfh. We found that most Nmb^+^ neurons are small-diameter DRG neurons as they are also peripherin^+^ (76.89 ± 6.62% Nmb^+^ neurons express peripherin; 51.53 ± 4.59% peripherin^+^ neurons express *Nmb*; Figure 5A-5D). In addition, about a quarter of Nmb^+^ neurons are large-diameter and Nfh^+^ (28.53 ± 3.23% Nmb^+^ neurons express Nfh; 28.73 ± 7.42% Nfh^+^ neurons express *Nmb*; Figure 5E-5H). Furthermore, we measured the size of Nmb^+^ DRG neurons and plotted their size distribution with regard to those of peripherin^+^ and Nfh^+^ neurons. Our data illustrates a skewed distribution in the size of Nmb^+^ neurons, with a majority of neurons showing a size distribution similar to peripherin^+^ neurons and a small proportion having larger areas comparable to Nfh^+^ neurons (Figure 5I). These results suggest that *Nmb* is expressed in both small and large diameter DRG neurons, but most Nmb^+^ neurons have small-diameter somata.

### *Nmb* is expressed in both peptidergic and non-peptidergic nociceptors

**Figure 6:**
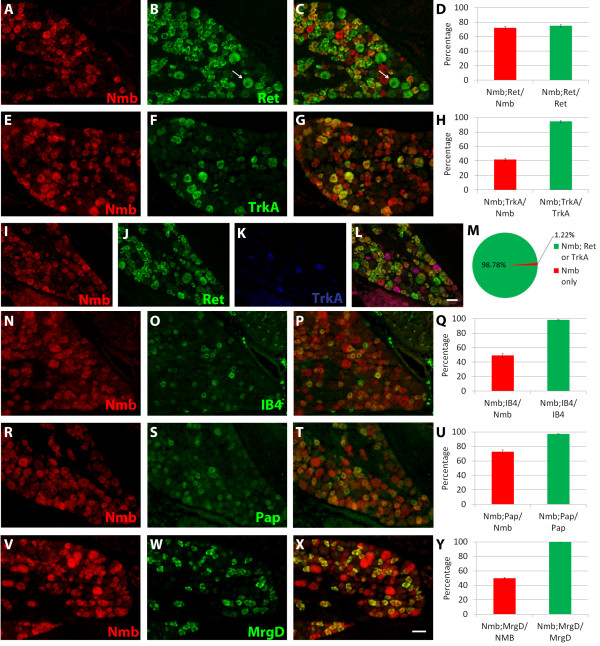
***Nmb*is expressed in peptidergic and nonpeptidergic nociceptors.** (**A**-**D**) FISH for *Nmb* (red) and *Ret* (green), a marker for small diameter nonpeptidergic nociceptors and RA mechanoreceptors. Arrow indicates a large diameter, Ret^+^ neuron, all of which are Nmb^-^. (**E**-**H**) FISH for *Nmb* (red) and *TrkA* (green), a marker for peptidergic nociceptors. (**I**-**M**) FISH for *Nmb* (red) and *Ret* (green), combined with immunostaining for TrkA (blue). Quantification shows the percentages of Nmb^+^ neurons which also express Ret or TrkA (green), or which express neither marker (red). (**N**-**Q**) FISH for *Nmb* (red) and binding of IB4 (green). (**R**-**U**) FISH for *Nmb* (red) combined with immunostaining for Pap (green). (**V**-**Y**) FISH for *Nmb* (red) and *MrgprD* (green). Scale bar = 50 μm.

As mentioned above, most small diameter DRG neurons are nociceptors. To address whether *Nmb* is expressed in pain- and itch-sensing DRG neurons, we first examined *Nmb* expression in two broadly defined classes of pain-sensitive neurons: peptidergic and nonpeptidergic nociceptors. During embryonic development, all nociceptors express TrkA, the neurotrophic receptor tyrosine kinase for nerve growth factor (NGF). In the first two postnatal weeks, however, DRG neurons that differentiate into nonpeptidergic nociceptors extinguish their expression of *TrkA* and express another receptor tyrosine kinase, Ret, while peptidergic nociceptors retain their expression of *TrkA* into adulthood [17,23]. We performed double FISH for *Nmb* and *Ret* or *TrkA* to determine which classes of nociceptors express *Nmb*. Almost all TrkA^+^ neurons (41.99 ± 2.01% of Nmb^+^ neurons express *TrkA*; 94.59 ± 1.14% of TrkA^+^ neurons express *Nmb*) and majority of Ret^+^ neurons (72.14 ± 2.54% of Nmb^+^ neurons express *Ret*; 75.04 ± 1.58% of Ret^+^ neurons express *Nmb*) express *Nmb* (Figure 6A-H). The Ret^+^ DRG neurons are comprised of both small-diameter nonpeptidergic nociceptors and larger-diameter rapidly adapting (RA) mechanoreceptors [24]. Interestingly, all large-diameter Ret^+^ RA mechanoreceptors are Nmb^-^ (arrow, Figure 6B6C), and the proportion of Ret^+^ neurons that co-express *Nmb* is very similar to the proportion of Ret^+^ DRG neurons that are nociceptors [17,25]. Additionally, we found that almost all Nmb^+^ neurons express either *Ret* or TrkA (98.78 ± 0.12% of total NMB^+^ neurons) when we combine double FISH for *Nmb* and *Ret* with immunohistochemistry for TrkA (Figure 6I6M). We also note that Nmb^+^/TrkA^+^ neurons are larger than Nmb^+^/Ret^+^ neurons. In short, these results indicate that *Nmb* is specifically expressed in both peptidergic and non-peptidergic nociceptors.

To further confirm the expression of *Nmb* in nonpeptidergic nociceptors we combined *Nmb* FISH with immunostaining for Pap or binding of isolectin-B4, markers that exclusively label nonpeptidergic nociceptors. *Nmb* was expressed in nearly all DRG neurons that bind IB4 (49.20 ± 2.98% of Nmb^+^ neurons bind IB4; 98.47 ± 0.25% of IB4-binding neurons express *Nmb*) or that are Pap^+^ (72.61 ± 2.79% of Nmb^+^ neurons express Pap; 97.28 ± 0.23% of Pap^+^ neurons express *Nmb*) (Figure 6N6U). Additionally, we examined the expression of *Nmb* in neurons that express the G-protein coupled receptor *MrgprD*, which comprise a large subset of nonpeptidergic nociceptors which mediate the sensation of mechanical pain [26]. *Nmb* was expressed in all DRG neurons which express *MrgprD* (49.89 ± 1.24% of Nmb^+^ neurons express MrgprD; 100 ± 0% of MrgprD^+^ neurons express Nmb) (Figure 6V6Y). Taken together, our data indicate that *Nmb* is expressed in nearly all peptidergic and nonpeptidergic nociceptors.

### *Nmb* is expressed in itch-sensing neurons

**Figure 7:**
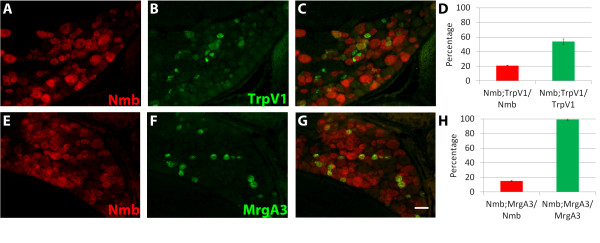
***Nmb*is expressed in itch-sensitive DRG neurons.** (**A**-**D**) FISH for *Nmb* (red) and *TrpV1* (green), a marker of neurons responding to noxious and histaminergic itch stimuli. (**E**-**H**) FISH for *Nmb* (red) and *MrgprA3* (green), a marker of neurons responding to nonhistaminergic itch. Scale bar = 50 μm.

Itch-sensing neurons have been suggested to be a subpopulation of nociceptors. We next asked whether *Nmb* is present in potential itch-sensing DRG neurons. The transient receptor potential channel TrpV1 was originally characterized as the channel mediating the burning sensation associated with capsaicin administration [27]. Additionally, TrpV1^+^ neurons have been shown to play important roles in the detection of noxious heat and mediation of itch sensation in response to histaminergic stimuli [28,29]. Double FISH revealed that *Nmb* is expressed in approximately half of TrpV1^+^ DRG neurons (20.94 ± 0.56% of Nmb^+^ neurons express *TrpV1*; 53.72 ± 3.66% of TrpV1^+^ neurons express *Nmb*) (Figure 7A-D).

To further investigate the expression of *Nmb* in itch-sensing DRG neurons, we performed double FISH for *Nmb* and *MrgprA3*. MrgprA3 is a G-protein coupled receptor which is expressed in a subset of small diameter DRG neurons and is activated by chloroquine, a non-histaminergic pruritogen [30]. Remarkably, we found that almost all MrgprA3^+^ DRG neurons co-express *Nmb*, even though MrgprA3 is expressed in only a small proportion of Nmb^+^ neurons(15.10 ± 0.93% Nmb^+^ neurons express *MrgprA3*; 99.19 ± 0.81% MrgprA3^+^ neurons express *Nmb*) (Figure 7E-H). In summary, our results suggest that *Nmb* is expressed in populations of DRG neurons that mediate itch sensation.

### *Nmb* is not expressed in cold-sensing and low-threshold mechanosensory neurons

**Figure 8:**
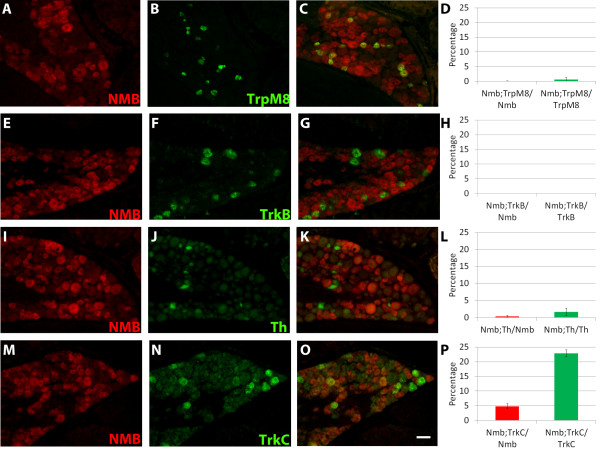
***Nmb*is not expressed in DRG neurons responding to innocuous thermal or mechanical stimuli.** (**A**-**D**) FISH for *Nmb* (red) and *TrpM8* (green), a marker of neurons responding to innocuous cold stimuli. (**E**-**H**) FISH for *Nmb* (red) and *TrkB* (green), a marker of Aδ LTMRs. (**I**-**L**) FISH for *Nmb* (red) and *TH* (green), a marker of a population of intermediately adapting C fiber LTMRs. (**M**-**P**) FISH for *Nmb* (red) and *TrkC* (green), a marker of slowly adapting mechanoreceptors and proprioceptors. Scale bar = 50 μm.

Subsets of small-diameter DRG neurons have been shown to respond optimally to various stimuli, including pruritogens, noxious thermal, mechanical, or chemical stimuli, innocuous temperature, or light touch. To test if the expression of *Nmb* is specific to pain- and itch-sensing neurons, we also performed double FISH for *Nmb* and molecular markers that identify temperature- or light touch-sensing neurons. One such marker is the transient receptor potential channel, TrpM8. TrpM8 has been shown to be important for the detection of environmental, but not noxious, cold (temperatures ~15°-26°C), and to be activated by chemicals which induce a cooling sensation, such as methanol and icilin [31]. Interestingly, expression of *Nmb* is almost completely non-overlapping with that of *TrpM8* (0.08 ± 0.08% Nmb^+^ neurons express *TrpM8*; 0.61 ± 0.61% of TrpM8^+^ neurons express *Nmb*) (Figure 8A-D).

To further test our hypothesis that *Nmb* is specifically expressed in pain- and itch- sensing neurons, we compared the expression of *Nmb* to markers of low-threshold mechanosensitive neurons, which mediate the sensation of light touch, texture, form, vibration, and body position. Low-threshold mechanosensory neurons contain several different groups of DRG neurons, each of which have distinct molecular and developmental profiles. We examined the expression of *Nmb* with regard to three classes of low-threshold mechanosensitive neurons: Aδ low-threshold mechanoreceptors (Aδ LTMRs), c-fiber low-threshold mechanoreceptors (C-LTMRs), and proprioceptors.

Aδ LTMRs, which are also known as D-hair, express the neurotrophic tyrosine kinase receptor TrkB postnatally [32]. We found that the expression of *TrkB* and *Nmb* mRNA is completely non-overlapping in juvenile mouse DRG neurons (0 ± 0% Nmb^+^ neurons express *TrkB*; 0 ± 0% TrkB^+^ neurons express *Nmb*) (Figure 8E8H). We also examined the overlap between *Nmb* and tyrosine hydroxylase (Th), a marker that is expressed postnatally in intermediately adapting, small-diameter C fiber LTMRs [32]. Even though most Nmb^+^ neurons have small-diameter somata, we found minimal overlap between *Nmb* and Th expression (0.37 ± 0.26% Nmb^+^ neurons express Th; 1.64 ± 1.09% Th^+^ neurons express *Nmb*) (Figure 8I8L). Moreover, we have shown in the previous section that *Nmb* is not expressed in large-diameter Ret^+^ neurons, which are Aβ RA mechanoreceptors (Figure 6C). Taken together, our results strongly suggest that *Nmb* is not expressed in LTMRs, which is consistent with our hypothesis that *Nmb* is expressed exclusively in pain- and itch- sensing neurons.

We next examined the expression of *Nmb* in neurons which express the neurotrophic receptor tyrosine kinase TrkC, which is commonly used as a marker of proprioceptive and Aβ slowly adapting mechanosensitive DRG neurons. We found that a very small percentage of Nmb^+^ neurons co-express *TrkC* (4.81 ± 0.93% Nmb^+^ neurons express *TrkC*; 22.86 ± 1.16% TrkC^+^ neurons express *Nmb*) (Figure 8M-8P). Attempts to further clarify whether *Nmb* is expressed in proprioceptors were unsuccessful, as another classical immunohistochemical marker of proprioceptors, parvalbumin, did not work in combination with our *in-situ* hybridization protocol. At present, we are uncertain about the functional identity of these Nmb^+^/TrkC^+^ neurons. Nevertheless, our hypothesis that *Nmb* is specifically expressed in pain- and itch- sensing neurons should still be true, as Nmb^+^/TrkC^+^ neurons are only a very small percentage of total Nmb^+^ neurons (less than 5%).

## Discussion

In this paper, we have presented evidence demonstrating that Grp, which has been proposed to act as a selective neurotransmitter of itching sensation from the DRG to the dSC, is also highly expressed in dSC neurons. In addition, we have found that Nmb, a homologue of Grp, is highly and selectively expressed in pain- and itch-sensing DRG neurons. Our anatomical characterization suggests that Grp can function intraspinally in dSC neurons in addition to its potential roles in DRG neurons and Nmb could play roles in pain- and itch-sensing neurons.

### *Grp* is highly expressed in dSC neurons

By employing multiple methods to detect *Grp* mRNA and protein, we found that *Grp* is robustly expressed in dSC neurons. Even though a previous publication suggests that no *Grp* mRNA is detected in adult mouse spinal cord by *in situ* hybridization [2], we detected abundant *Grp* mRNA in both juvenile and adult mouse dorsal spinal cord using *in situ* hybridization, RT-PCR, and real time PCR. We cannot make a comparison or suggest possible explanations for these different results because the *in situ* hybridization data of *Grp* in the previous publication is not shown. Nevertheless, our results are in line with the P4 *in situ* hybridization data of the Allen Mouse Spinal Cord Atlas, suggesting that the expression pattern of *Grp* in DRG and SC neurons is fairly stable from early postnatal days to adulthood [12]. Our results are also consistent with the P7 and adult *Grp* expression pattern provided by the St. Jude Brain Gene Expression Map (BGEM) using a radioactive *in situ* hybridization method [33]. Furthermore, *Grp* mRNA was shown to be highly expressed in dSC in adult rat [6]. Taken together, our results and information from public databases strongly support the conclusion that *Grp* mRNA is highly expressed in postnatal dSC neurons.

In agreement with the low level of *Grp* mRNA found in DRG neurons (Figure 1B and references [6,12] ), we only detected a small number of Grp^+^ DRG neurons using immunostaining (Figure 3A3G). The Grp^+^ neurons we detected are much less than that found in a previous publication (8.6% of lumbar DRG neurons [2]). This difference could be due to different fixation conditions. Though we detected a robust level of Grp peptide in dSC neurons using immunostaining (Figure 3K, O, Q, S), we barely detect any Grp in dorsal root nerve (Figure 3V), the only pathway between DRG neurons and dSC, indicating that the amount of Grp synthesized and transported from DRG to dSC is minimal and below the detection level of our methods.

To further confirm the source of dSC Grp, we also conducted the dorsal root rhizotomy. Since proteins or peptides derived from DRG neurons need to be transported through the dorsal root, their presence in the spinal cord should dramatically decrease following central root rhizotomy. On the other hand, reduction of proteins or peptides that are primarily synthesized in dSC neurons should not be as dramatic. Indeed, we found ~80% decrease of markers originating from DRG neurons, such as Cgrp and Pap, and only found ~ 35% decrease of a spinal neuron marker, Pkcγ. Importantly, the change of dSC Grp is significantly different from DRG neuron derived markers but similar to that of Pkcγ (Figure 4S), suggesting that the majority of Grp is synthesized locally in dSC neurons. This result is consistent with the expression pattern of *Grp* mRNA. Taking all data into consideration, our results strongly argue that a majority of dSC Grp is synthesized locally in spinal cord neurons.

### *Nmb* is specifically expressed in pain- and itch-sensing DRG neurons

In contrast to *Grp*, we found that the other known mammalian bombesin peptide, *Nmb*, is highly expressed in DRG neurons. By co-staining with molecular markers for different modalities of somatosensory neurons, we found that *Nmb* is selectively expressed in pain- and itch-sensing neurons. This specific expression pattern suggests that Nmb may play a role in these neurons. In addition to its potential role as a neurotransmitter in pain- and itch-sensing neurons, as discussed below, another interesting possibility is that DRG neuron derived Nmb may be released by peripheral axons and functions peripherally. In fact, we detected some expression of *Nmbr* in mouse glabrous skin by *in situ* hybridization (data not shown). Moreover, *Nmbr* mRNA levels were found to be increased following mechanical wounding and bombesin can promote keratinocyte mitosis and migration *in vitro*[34]. These observations and our anatomical characterization raise the intriguing possibility that Nmb could be released from free nerve terminals upon injury to promote skin regeneration and wound healing.

### Neurotransmitter for itch-sensing somatosensory neurons

Which chemical do itch-sensing somatosensory neurons use as the pruritogenic neurotransmitter? This is an important question as a pruritogenic specific neurotransmitter would be a potential target for treating patients with pruritic disease. Some recent studies have proposed glutamate and Grp as two candidate neurotransmitters for itch-sensing somatosensory neurons [2,14-16]. One anatomical requirement for a chemical to function as the pruritogenic neurotransmitter is that it is synthesized in DRG neurons, transported to dSC, and activates its receptor, which is expressed in postsynaptic neurons. We found that *Grp* mRNA is expressed at a low level in DRG neurons and a small amount of Grp is transported from DRG neurons to dSC. Interestingly, we found that a high level of Grp is synthesized in dSC neurons, which may also contribute to normal itch perception by modulating the spinal cord local circuits. Future molecular and behavior analysis with *Grp* null or DRG vs. dSC specific *Grp* knockout mice will help to address the expression and function of DRG neuron and dSC neuron derived Grp.

On the other hand, Nmb, the other mammalian bombesin-related peptide, could be an interesting candidate neurotransmitter to modulate pain and itch sensation, given its high and selective expression in DRG neurons. Though we did not detect significant amount of *Nmbr* mRNA in dSC using *in situ* hybridization at P21, intrathecal injection of Nmb induces itching behavior [11]. This response may be conducted through Nmbr, which could be expressed in dSC at a low level. Alternatively, Nmb could be acting though Grpr, which Nmb can bind to with a low affinity [5]. Thus, a good antibody against Nmbr or behavior analysis with *Nmb* and *Nmbr* knockout mice in the future will help to address the localization of Nmbr and physiological roles of DRG neuron derived Nmb.

## Conclusions

We have found that *Grp* transcript is highly expressed in dSC neurons and the majority dSC Grp is synthesized locally. On the other hand, *Grp* transcript is expressed at a low level in DRG neurons, and that the contribution of DRG neuron-derived Grp to the total amount of Grp observed in the dSC is relatively small. In addition, we have shown that *Nmb*, the other mammalian bombesin-related peptide, is highly expressed in pain- and itch-sensing DRG neurons. Taken together, our results suggest that intraspinally synthesized Grp may have a local function in addition to potential functions of DRG neuron-derived Grp and that *Nmb* may play a role in itch- or pain-sensing neurons. This anatomical characterization of Grp and *Nmb* will improve our understanding about roles of Grp and Nmb in mediating itch sensation.

## Materials and methods

### Animals

Mice were raised in a barrier facility in the Hill Pavilion, University of Pennsylvania. All animal procedures were conducted according to animal protocols approved by the Institutional Animal Care and Use Committee (IACUC) of the University of Pennsylvania. For *in-situ* hybridization and antibody characterization studies, six P14-P21 wild type mice of both sexes with a mixed CD1/C57Bl/6 J background were used. For RT-PCR experiments, four 4–6 month wild type mice of both sexes with a mixed CD1/C57Bl/6 J background were used. For dorsal rhizotomy experiments, we used *2* month old C57Bl/6 J mice of either sex and the survival surgery was conducted in Shriners Hospitals Pediatric Research Center, Temple University. All surgical and postoperative procedures were performed in accordance with Temple’s Institutional Animal Care and Use Committee and National Institutes of Health guidelines.

### *In situ* hybridization

DIG-labeled riboprobes were synthesized using a DIG RNA labeling kit (Roche, 11175025910). Template for Nmb probe was amplified from a mouse DRG cDNA library (BD, Ref#630022) and subcloned into vector pGEM-T Easy (Promega, A1360). Mouse IMAGE clones for Grp (GenBank: BC024515), Grpr (GenBank: BC113145), and Nmbr (GenBank: BC119237) were purchased from Open Biosystems, and PCR products were subcloned into pGEM-T Easy. Primers used to amplify cDNA were: Nmb (5’-GGCAAGCAGGGAGCTCTT-3’ and 5’-CTGGTGACCCAACCAGAA-3’), Grp (5’-CACGGTCCTGGCTAAGATGTAT-3’ and 5’-CCAGTAGAGTTGACGTTTGCAGA-3’), Nmbr (5’-AGGTCTCTCTCCAACCTCTCCT-3’ and 5’-ACCAGAACAATCTTAGCCAGGCG-3’), and Grpr (5’-ATGGCTCCAAATAATTGTTCCCA-3’ and 5’-TTTAGTCTAGACATACCCCTCAT-3’). FITC-labeled probes for c-Ret, TrkB, TrkC, TrpV1, MrgprA3, MrgprD, and TrpM8 were generated as previously described [23,35].

Intact lumbar spinal column was dissected from euthanized wild-type P14-P21 mice and rapidly frozen in OCT on a dry-ice/ethanol bath. 20 μm cryosections were collected on Superfrost Plus slides (Fisher, 22-034-979) and allowed to dry for at least 2 hours at room temperature before *in-situ* hybridization. All steps prior to hybridization were carried out under RNase free conditions. Cryosections were immersion-fixed in freshly made 4% PFA in PBS for 20 minutes at room temperature. Slides were then washed in fresh-DEPC PBS (1:1000 DEPC in PBS immediately before use), followed by wash in DEPC-pretreated PBS (1:1000 DEPC in PBS overnight (O/N), followed by autoclaving). An antigen retrieval step, often used for immunohistochemistry, was found to increase the signal of many probes. Citric acid buffer (10 mM citric acid, 0.05% Tween-20, pH6.0) was boiled in a microwave, and DEPC (1:1000) was added to freshly boiled solution. Slides were immersed in solution in a 95°C waterbath for 20 minutes, and then allowed to cool at room temperature for 30 minutes. Sections were then washed in DEPC pretreated PBS (1X 5 minutes), incubated in Proteinase K (25 μg/mL in DEPC-pretreated H_2_O) for five minutes, followed by washes in fresh-DEPC PBS (1X 5 minutes) and DEPC pre-treated PBS (1X 5 minutes). Sections were then acetylated at room temperature for ten minutes in freshly made acetylation solution (0.1 M triethanolamine, 0.25% acetic anhydride in DEPC pre-treated H_2_O). Slides were then prehybridized in hybridization buffer (50% formamide, 5XSSC, 0.3 mg/mL yeast tRNA, 100 μg/mL heparin, 1X Denhardt’s, 0.1% Tween-20, 0.1% CHAPS, 5 mM EDTA in RNase free H_2_O) at 62°C in a humidified chamber for 30 minutes. Following prehybridization, excess hybridization buffer was removed from slides and 1-2 ng/μl of DIG and/or FITC labeled riboprobe diluted in hybridization buffer was placed on the slide. Slides were incubated O/N under Parafilm coverslips at 62°C. Slides were then washed in 0.2X SSC at 68°C (1X 15 minutes, 2X 30 minutes).

For colorimetric reaction, slides were blocked in PBT (PBS, 0.1% TritonX-100) and 20% lamb serum at room temperature for one hour. Sections were then incubated with AP-conjugated anti-DIG antibody (1:1000; Roche, 11093274910) in blocking buffer O/N at 4°C. Slides were washed in PBT (3X 10 minutes) and incubated O/N in darkness in alkaline phosphatase buffer (100 mM Tris pH9.5, 50 mM MgCl_2_, 100 mM NaCl, 0.1% Tween-20, 5 mM levamisole, 0.34 mg/mL 4-Nitro blue tetrazolium (NBT)(Roche, 11383213001), 0.17 mg/mL 5-bromo-4-chloro-3-indolyl-phosphate(BCIP)(Roche, 1138221001)). Following color reaction, slides were rinsed repeatedly in PBS and then fixed for 20 minutes in 4% PFA in PBS at room temperature. Slides were then repeatedly rinsed in ddH_2_O, dried at 37°C for 1 hour, dehydrated in xylenes (3X 2 minutes), and coverslipped with Permount (Fisher, SP15).

For double fluorescent in-situ hybridization (FISH), slides were blocked for one hour at room temperature with 0.5% Blocking Reagent (Roche, 11096176001) in PBS. Sections were incubated in anti-FITC-POD (1:100 in .5% Blocking Reagent; Roche, 11426346910) O/N at 4°C. Slides were then washed in PBT (3X10 minutes) and incubated in 0.1% BSA in PBS for 15 minutes. FITC riboprobes were then developed using the TSA Plus system (Perkin Elmer, NEL741001KT), by diluting fluorescein tyramide into 1X amplification buffer (1:100) and incubating slides in working solution for 10–15 minutes, followed by washes in PBS (3X 10 minutes). Slides were then blocked in PBT containing 20% lamb serum for 1 hour at room temperature, and incubated O/N at 4°C with AP-conjugated anti-DIG antibody (1:500 in PBT +20% lamb serum). Slides were washed in TNT (100 mM Tris–HCl, 150 mM NaCl, 0.05% Tween-20, pH7.5) (3X 10 minutes), then in detection buffer (100 mM Tris–HCl, 100 mM NaCl, 10 mM MgCl_2_, pH8.0) (2X 10 minutes). DIG-labeled riboprobes were then developed using HNPP/Fast Red TR system (Roche, 11758888001). Sections were incubated in detection solution (10μL HNPP stock solution, 10μL of 25 mg/mL FastRed per 1 mL of detection buffer, filtered through 0.2 μM nylon filter) (3x30 minutes), with TNT rinses between incubations. Slides were then rinsed in PBS and mounted with Flourmount (Southern Biotech, 0100–01).

For FISH combined with immunofluorescence, normal hybridization procedure was followed, using DIG-labeled probe. After 0.2X SSC washes, sections were blocked for one hour in PBT containing 20% lamb serum. Sections were then incubated with AP-conjugated anti-DIG (1:500) and primary antibody at the appropriate dilution (described below) at 4°C O/N in 20% lamb serum blocking solution. Slides were washed in PBT (3X 10 minutes), then incubated in species appropriate Alexa 488 conjugated secondary antibody (1:500 in 5% lamb serum in PBT) for one hour at RT. HNPP/FastRed detection was then performed as described above, beginning with initial TNT washes.

### Immunohistochemistry

For characterization of anti-bombesin antibody, P14-P21 mice were deeply anesthetized with CO_2_ and perfused with 4% PFA in PBS. Intact lumbar spinal columns were dissected and post-fixed for 2–4 hours in 4% PFA in PBS at 4°C, cryoprotected in 30% sucrose in PBS O/N at 4°C, and embedded in OCT. 20 μm cryosections of lumbar spinal cord and DRG were collected on Superfrost Plus slides, and allowed to dry at room temperature for at least two hours. For dorsal rhizotomy samples, 30 μm free floating cryosections were collected in PBT and processed for immunohistochemistry in solution. Sections were washed in PBT (3X 10 minutes), and then blocked in PBS containing 5% lamb serum and 0.3% TritonX-100 for 1 hour at room temperature. Primary antibodies were diluted in the same buffer, and incubated O/N at 4°C, then washed in PBT (3X 10 minutes). Secondary antibodies were incubated in blocking buffer at 1:1000 dilution for one hour at room temperature. Slides were then washed in PBT (3X 10 minutes) and mounted with Flourmount. Primary antibodies used include rabbit anti-bombesin (1:1000; ImmunoStar, 20073), chicken anti-Gfp (1:2000; Aves, GFP-1020), guinea pig anti-Cgrp (1:250; Bachem, T-5053), mouse anti-Pkcγ (1:50, Invitrogen, 13–3800), chicken anti-Pap (1:1000; Aves, PAP), rabbit anti-peripherin (1:1000; Millipore, AB1530), rabbit anti-Nfh (1:2000; Sigma, N4142), rabbit anti-TrkA (1:1000; Millipore, 06–574), rabbit anti-tyrosine hydroxylase (1:100; Millipore, AB152), mouse anti-NeuN (1:1000, Millipore, MAB377), and Alexa 488 conjugated IB4 (1:200; Invitrogen, I21411). Secondary antibodies used were Alexa 488, Alexa 594, or Alexa 647 conjugated goat anti-rabbit antibody, Alexa 488 conjugated goat anti-mouse antibody, Alexa 488 conjugated goat anti-chicken antibody, and Alexa 488 conjugated goat anti-guinea pig antibody. All secondary antibodies were purchased from Invitrogen.

### Peptide preabsorption

Anti-bombesin and anti-Cgrp antibodies at the concentrations described above were added to PBS containing 5% lamb serum, 0.3% TritonX-100, and bombesin peptide (50 μg/mL; American Peptide Company, 16-7-10A) or GRP peptide (50 μg/mL; American Peptide Company, 62-3-10) and incubated O/N at 4°C with gentle agitation. Solutions were centrifuged at high speed (~16,000 x g) for ten minutes, and supernatant was used for immunohistochemistry, as described above.

### Reverse transcriptase PCR

4–6 month old wild type mice were deeply anesthetized with CO_2_, transcardially perfused with sterile, RNAse free, ice-cold PBS, then decapitated. Lumbar and thoracic DRGs and dorsal spinal cord were dissected under RNase free conditions and rapidly frozen on dry ice. RNA was isolated using the GeneJet RNA Purification Kit (Fermentas, K0731), and cDNA was synthesized with oligo-dt primers using the SuperScript First-Strand Synthesis system (Invitrogen, 11904018). PCR was performed on cDNA synthesized from DRG or dorsal spinal cord cDNA with primers for Nmb (5’- CCGAGGGACCAGAGACTACA-3’ and 5’-ACTTCACCAGGGAAGCAAGA), Grp (5’-CACGGTCCTGGCTAAGATGTAT-3’ and 5’-CCAGTAGAGTTGACGTTTGCAGA-3’), Nmbr (5’-AGGTCTCTCTCCAACCTCTCCT-3’ and 5’-ACCAGAACAATCTTAGCCAGGCG-3’), Grpr (5’-ATGGCTCCAAATAATTGTTCCCA-3’ and 5’-TTTAGTCTAGACATACCCCTCAT-3’), and Gapdh (5’-GGTGAAGGTCGGTGTGAACG-3’ and 5’-CTCGCTCCTGGAAGATGGTG-3’).

### Real time PCR

*Grp* mRNA from DRG or dSC were measured by quantitative real-time PCR under the ABI 7500 Fast Real-Time PCR system (Applied Biosystems, Foster City, CA), with *Gapdh* as an internal reference gene. The reaction mixture contained 250 nM of each primer, 1 × SYBR Green PCR Master Mix (ABI), and 1 μl of cDNA. Relative concentration of *Grp* present in each cDNA sample was calculated by the comparative C(T) method [36]. Real time PCR was performed on cDNA isolated from three adult wild type mice, and was repeated four times per sample. Primers used were Grp primers listed above, and Gapdh (5’-TCGGTGTGAACGGATTTGGC-3’and 5’-TCCCATTCTCGGCTTGACT-3’).

### Plasmid construction

Plasmids were constructed to express bombesin, Grp, or Nmb fused to the C terminus of EGFP. The coding regions of the peptides were constructed by annealing oligonucleotides coding for the open reading frame of each peptide with EcoRI (5’) and SalI (3’) sticky ends. Primers used for this procedure were: bombesin (5-AATTCCAGCAGAGGCTGGGGAATCAGTGGGCAGTGGGTCACTTGATGTGAG-3’ and 5’-TCGACTCACATCAAGTGACCCACTGCCCACTGATTCCCCAGCCTCTGCTGG-3’) Grp (5’-AATTCATGTATCCGCGCGGCAGTCACTGGGCTGTGGGACACTTAATGTGAG-3’ and 5’-TCGACTCACATTAAGTGTCCCACAGCCCAGTGACTGCCGCGCGGATACATG-3’) and Nmb (5’AATTCGGCAACCTCTGGGCGACCGGTCACTTCATGTGAG-3’ and 5’-TCGACTCACATGAAGTGACCGGTCGCCCAGAGGTTGCCG-3’). Oligonucleotides were phosphorylated with T4 polynucleotide kinase (PNK) (NEB, M0201) for 20 minutes at 37°C, and then PNK was heat inactivated at 65°C for 20 minutes. Phosphorylated oligonucleotides were annealed by heating to 95°C for five minutes, then allowing them to cool to room temperature. Annealed oligonucleotides were then ligated into pPMS93 (a gift from Dr. Jeremy Nathans lab at Johns Hopkins University, CMV promoter to drive the expression of EGFP C-terminal fusion protein) pre-digested with EcoRI and SalI. Ligated plasmids were transformed into DH5-α, and grown overnight at 37°C on agarose plates supplemented with 100 μg/mL carbenicillin. Individual colonies were then selected and grown overnight in 2XYT containing 100 μg/mL carbenicillin, and then mini-prepped (Fermentas, K0503). Correct insertions were confirmed by DNA sequencing.

### Cell culture and immunocytochemistry

QBI HEK293 cells were grown on 12 mm circle coverslips in HEK293 growth media (10% FBS (Invitrogen, 10082147), 1% Penicillin/Streptomycin (Invitrogen, 15140122) in DMEM (Invitrogen, 11965084)) in 6-well tissue culture plates. Coverslips were coated with 100 μg/mL poly-D-lysine solution to help cell adhesion. Individual cultures were transfected with 1 μg/mL of pRK5-EGFP, pPMS-bombesin, pPMS-Grp, or pPMS-Nmb EGFP fusion plasmid with Lipofectamine LTX (Invitrogen, 15338100). Eighteen to twenty four hours after transfection, coverslips were then rinsed gently with PBS and fixed in 4% PFA in PBS for 1.5 hours at room temperature. Coverslips were then processed for immunohistochemistry as described above.

### Dorsal root rhizotomy and postoperative procedures

The surgical procedure was standard [37]. Mice were anesthetized with an intraperitoneal injection of xylazine (8 mg/kg) and ketamine (120 mg/kg). Supplements were given during the procedure as necessary. A 2- to 3-cm-long incision was made in the skin of the back; the spinal musculature was reflected; and the L5 spinal cord segments were exposed by hemi-laminectomies. The cavity made by the laminectomies was perfused with warm sterile Ringer's solution. A small incision was made in the dura overlying the L3-L5 dorsal roots; a fine spring scissor (501778, World Precision Instruments) was introduced subdurally and L4, L5, and L6 dorsal roots were cut. The laminectomy site was covered with a piece of thin synthetic matrix membrane (Biobrane, Bertek Pharmaceuticals), which was then stabilized with a layer of thicker artificial dura (Gore Preclude MVP Dura Substitute, W.L. Gore and Associates). Animals are given subcutaneous injections of lactated Ringer's solution to prevent dehydration and are kept on a heating pad until fully recovered from anesthesia. Buprenex is given as post-operative analgesia (0.05 mg/kg S.C. every 12 hours for 2 days). On the 15^th^ day after dorsal rhizotomy, mice were perfused transcardially with 0.9% heparinized saline solution followed by 4% paraformaldehyde in PBS.

### Imaging

All images were acquired on a Leica DM5000B or Leica TCS SP5 II and processed using Adobe Photoshop CCS version 12.0.

### Quantification and statistics

For dorsal rhizotomy experiments, adjacent sections of the spinal cord lumbar enlargement were used for different marker staining. Images of each dorsal horn of individual sections were acquired using identical exposure conditions. Using ImageJ, the bombesin reactive and Cgrp, Pkcγ, or Pap (costain) reactive regions of dorsal spinal cord were outlined. Pixel counts for each fluorescence intensity value (0–255) within the outlined region were generated by ImageJ. In addition, background pixel intensity values were generated from a non-reactive area of the dorsal spinal cord for each image. Background pixel counts were clustered around low intensity values and showed a normal distribution, and the threshold for background fluorescence cutoff was established as the first highest intensity value that had a pixel count of zero. To calculate total fluorescence intensity, the pixel count for each intensity above background level was multiplied by its intensity, and the results of these calculations were summed. In cases where the immunostaining signal was greatly reduced on transected side, the stain area was approximated based on the control side. The difference between the control and transected side was calculated as percentage change in fluorescent intensity ([fluorescent intensity transected/fluorescent intensity control]*100). This value was calculated for three sections per marker per animal. The average and SEM for each marker across all sections was calculated, and P-values were calculated using a two-tailed student’s t-test.

### Cell size calculations

ImageJ software was used to calculate size of DRG neurons. All images were taken at 20x magnification. To avoid bias in counting, the first 25 positive cells in each image from left to right were counted. In total, 225 cells per marker were counted (n = 3 animals, 9 DRG per animal). Using ImageJ, cells were outlined using the lasso tool and the area was obtained. For graphical representation, cells were divided into 100um^2^ bins.

### Expression profile quantification

Expression profile of Nmb^+^ DRG neurons was evaluated by counting the number of cells that were positive for Nmb, co-staining markers, or both in 3 wild-type P14-P21 animals (6–8 lumbar DRG sections per animal). Total number of positive cells in each category was calculated for each animal, and the percentages of double positive neurons with regard to total Nmb^+^ neurons (Nmb^+^; costain^+^ /total Nmb^+^) or to total co-staining-marker-positive neurons (Nmb^+^; costain^+^ positive/total costain^+^) were calculated. The average and SEM of these percentages between animals were then calculated.

## Abbreviations

CNS, Central nervous system; DRG, Dorsal root ganglion; dSC, Dorsal spinal cord; Grp, Gastrin releasing peptide; Grpr, Gastrin releasing peptide receptor; LTMR, Low-threshold mechanoreceptor; Nfh, Neurofilament heavy chain; Nmb, Neuromedin-B; Nmbr, Neuromedin-B receptor; TG, Trigeminal ganglion; Th, Tyrosine hydroxylase; RT-PCR, Reverse transcriptase PCR.

## Competing interests

The authors declare no competing interests.

## Authors’ contributions

MF generated EGFP fusion constructs and performed *in situ* hybridization, RT-PCR, FISH, 293 cell, DRG, and SC immunostaining, imaging, quantification, and statistical analysis; DR helped to generate the EGFP fusion constructs, performed HEK 293 T cell transfection, and quantified cell size and number of NMB^+^ neurons and other markers; SBH and YS performed the dorsal root rhizotomy; JZ performed real time RT-PCR; MF, YS, and WL designed experiments and finished the final draft of the manuscript. All authors read and approved the final manuscript.
